# Book Review

**Published:** 1946

**Authors:** 


### Reviews of Books

**Demonstrations of Physical Signs in Clinical Surgery.** By Hamilton Bailey, F.R.C.S. Tenth Edition. Pp. xii. +372. Illustrated. Bristol: John Wright & Sons Ltd. 1946. Price 30s.—A medical book which has run into ten editions in less than twenty years, which has been, or is being translated into seven languages, carries its own commendation. The immediately outstanding value of this, as of the older editions, is due to its lavish display of illustrations. These are well chosen and well displayed, often in colour, and they number 573, nearly two to a page. Nowhere else, as far as we are aware, can one find the like. Nor is it fair to say that the conditions illustrated are so rare that they would never be seen in ordinary practice. But if it is the pictures that catch the eye, the text is equally valuable, thoroughly up to date, and bearing evidence of a wide clinical experience.

**Leprosy.** By Sir Leonard Rogers, K.C.S.I., C.I.E., M.D., F.R.C.P., F.R.C.S., F.R.S., I.M.S., and Ernest Muir, C.I.E., M.D., F.R.C.S. Third Edition. Pp. xii. +280. Illustrated. Bristol: John Wright & Sons Ltd. 1946. Price 25s.—This is the third edition of a book which has long since taken its place as the most authoritative work upon the subject in small compass in the English language. This edition will do nothing to lessen the position won by its predecessors. The authors speak out of a long experience with authority but are never dogmatic. The whole field is covered adequately though naturally briefly. The fascinating story of the history of the disease is related in such an interesting manner that the reviewer read every word of it. At first sight he was inclined to think that possibly a little too much space had been allotted to questions of history at the expense of a more detailed description of the treatment, but upon re-reading this section he came to the conclusion that this was an unfair criticism and that the doctor desirous of knowing how to deal with any particular case of leprosy can find here all he need know, and should he desire further information, one very valuable feature of the book is the exhaustive bibliography provided. The illustrations are very good and excellently reproduced. The format is up to the standard we expect of the publishers, and the whole production a credit alike to authors and producers.

**Anatomy of the Bronchial Tree.** By R. C. Brock, M.S., F.R.C.S. Pp. vi. +96. Illustrated. London: Oxford University Press (Geoffrey Cumberlege). 1946. Price 42s.—This book is a reprint of articles published in the *Guy's Hospital Reports*. It embodies the results of very careful and extensive research into the anatomy of the bronchi, with particular reference to the part of the lungs associated with each branch and the surface markings and surgical approach. It is beautifully reproduced, with lavish diagrams (many in colour) and plates showing dissections and X-ray films—in all, more than twice as many illustrations as pages of text. It is certainly a book that every consultant must possess whose work involves the chest.

**Textbook of Psychiatry.** By D. K. HENDERSON, M.D., F.R.F.P.S., and R. D. GILLESPIE, M.D., D.P.M. Sixth Edition. Pp. 732. Illustrated. London: Oxford University Press. 1944. Price 25s.—Since the first edition of this book was published in 1927, it has remained the most important text-book of psychiatry written and published in England. In this, the latest edition, the lay-out is much the same as before, but in certain details it has been brought up to date and the modern methods of physical treatment are described in some detail. The psycho-pathology remains very much as before: the authors adhere to the biological view point of Adolf Meyer and the teaching of his followers in the American school. The text-book is sound, but students will not find it any easier to read than the previous editions, and perhaps the case-histories could have been shortened and clarified with advantage. The bibliography is probably neither as detailed nor as comprehensive as one would like to see in the principal standard text-book.

